# Draft Genome Sequence of *Komagataeibacter intermedius* Strain AF2, a Producer of Cellulose, Isolated from Kombucha Tea

**DOI:** 10.1128/genomeA.01404-15

**Published:** 2015-12-03

**Authors:** Renato Augusto Corrêa dos Santos, Andresa Aparecida Berretta, Hernane da Silva Barud, Sidney José Lima Ribeiro, Laura Natalia González-García, Tiago Domingues Zucchi, Gustavo H. Goldman, Diego M. Riaño-Pachón

**Affiliations:** a Laboratório Nacional de Ciência e Tecnologia do Bioetanol (CTBE), Centro Nacional de Pesquisa em Energia e Materiais (CNPEM), Campinas, São Paulo, Brazil; b Faculdade de Ciências Farmacêuticas de Ribeirão Preto, Universidade de São Paulo, Ribeirão Preto, São Paulo, Brazil; c Laboratório de Pesquisa, Desenvolvimento e Inovação, Apis Flora Industrial e Comercial Ltda., Ribeirão Preto, São Paulo, Brazil; d Instituto de Química, Universidade Estadual Paulista, Araraquara, São Paulo, Brazil; e Departamento de Ciencias Biológicas, Universidad de los Andes, Bogotá, Colombia; f AGRIVALLE, Salto, São Paulo, Brazil

## Abstract

Here, we present the draft genome sequence of *Komagataeibacter intermedius* strain AF2, which was isolated from Kombucha tea and is capable of producing cellulose, although at lower levels compared to another bacterium from the same environment, *K. rhaeticus* strain AF1.

## GENOME ANNOUNCEMENT

*Komagataeibacter intermedius* AF2, previously known as *Gluconacetobacter intermedius*, is a Gram-negative rod isolated from Kombucha tea. Briefly, for the isolation of *K. intermedius* AF2, 1 ml of Kombucha tea was subjected to serial 10-fold dilutions in 0.85% sterile NaCl solution. Aliquots of each dilution were plated on petri dishes containing Hestrin and Schramm (HS) medium. The plates were incubated aerobically at 30°C for 5 days. Suspected colonies were seeded in HS medium and incubated again as described above. After the incubation period, the colonies were transferred to test tubes (20 × 150 mm) containing HS broth and incubated aerobically at 30°C for five to seven days, in order to evaluate the production of cellulose, which can be easily observed on the surface of the culture medium. The AF2 isolate produced 1.41 g/L of cellulose, whereas *K. rhaeticus* strain AF1, isolated at the same time form the same environment, produced cellulose at higher levels (1).

Here, we present the genome sequence of *K. intermedius* strain AF2. This genome was sequenced on the Illumina HiSeq2000 system, generating 45,480,010 paired-end reads of 100 bp (insert size, 250 bp). The reads were preprocessed with Trimmomatic (2), resulting in 34,345,171 paired-end reads. Digital normalization was carried using Khmer (3), resulting in 482,978 reads, which were used to assemble contigs with SPAdes (4), using the best *k*-mer size chosen using KmerGenie (5). The post-assembly genome-improvement toolkit (PAGIT) was used to close gaps and correct substitution and insertion/deletion errors (6). An additional scaffolding step was carried out in SSPACE (7), followed by gap filling with GapFiller (8). The final assembly has a total length of 4,465,062 bp and an *N*_50_ of 70,565 bp, represented by 268 scaffolds. The average G+C content of the genome is 61.35%, which is similar to related species: *K. rhaeticus* AF1 (GC: 62.44%) (1); *K. xylinus* NBRC 3288 (GC: 60.92%) (9); *K. hansenii* (GC: 59.0%) (10); *K. europaeus* 5P3 (GC: 61.2%); *K. oboediens* 174Bp2 (GC: 61.3%) (11); and *Gluconacetobacter diazotrophicus* PaI 5 (GC: 66.19%) (12). Gene prediction was carried out with the Prokka Pipeline (13) using a nonredundant database of proteins in *Acetobacteriaceae* as the first annotation source. A total of 4,232 genes were identified, including 4,145 protein-encoding genes, 11 rRNA genes, 64 tRNA genes, 303 signal peptide genes, 1 tmRNA gene, and 11 ncRNA genes. Gene content is similar to related species: *K. rhaeticus* (3,460 genes), *K. xylinus* (3,195), *K. hansenii* (3,308), *K. medellinensis* (3,195), *K. europaeus* 5P3 (3,586), *K. oboediens* 174Bp2 (3,601), and *G. diazotrophicus* (3,864). A search against the UniProt database revealed 3,641 protein-encoding genes with strong sequence similarity hits to proteins in that database. The current genome assembly provides a preliminary landscape of the genomic and metabolic capabilities of *K. intermedius* strain AF2 and will provide insights about the molecular mechanisms involved in high and low cellulose production in this genus.

### Nucleotide sequence accession numbers.

This whole-genome shotgun project has been deposited at DDBJ/EMBL/GenBank under the accession number JUFX00000000. The version described in this paper is the second version, JUFX02000000.
